# Distant Organ Damage in Acute Brain Injury

**DOI:** 10.3390/brainsci10121019

**Published:** 2020-12-21

**Authors:** Natalia Rachfalska, Zbigniew Putowski, Łukasz J. Krzych

**Affiliations:** Department of Anaesthesiology and Intensive Care, Faculty of Medical Sciences in Katowice, Medical University of Silesia, 40-055 Katowice, Poland; n.rachfalska@gmail.com (N.R.); putowski.zbigniew@gmail.com (Z.P.)

**Keywords:** acute brain injury, organ dysfunction, critical illness, systemic response

## Abstract

Acute brain injuries pose a great threat to global health, having significant impact on mortality and disability. Patients with acute brain injury may develop distant organ failure, even if no systemic diseases or infection is present. The severity of non-neurologic organs’ dysfunction depends on the extremity of the insult to the brain. In this comprehensive review we sought to describe the organ-related consequences of acute brain injuries. The clinician should always be aware of the interplay between central nervous system and non-neurological organs, that is constantly present. Cerebral injury is not only a brain disease, but also affects the body as whole, and thus requires holistic therapeutical approach.

## 1. Introduction

**Figure 1:**
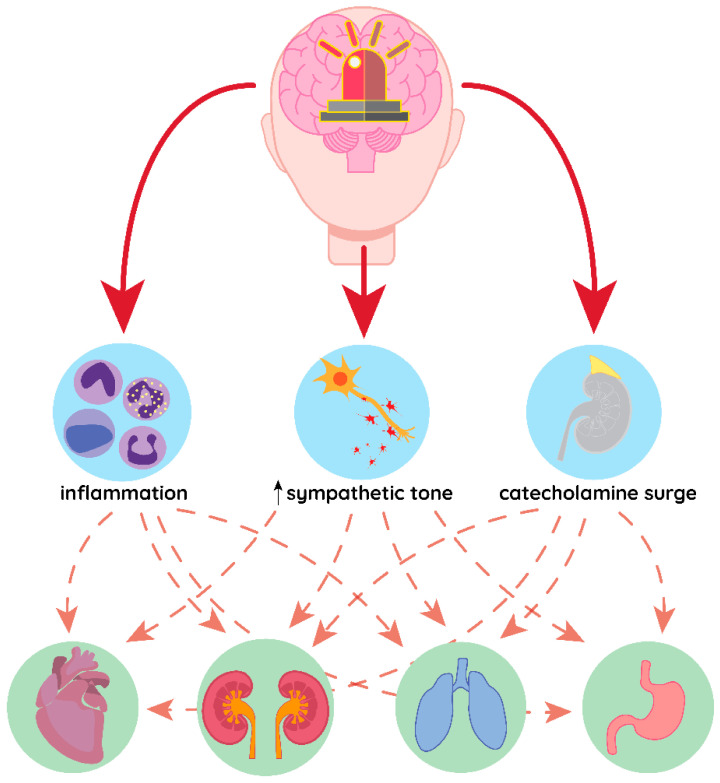
Common pathomechanism of distant organ damage in acute brain conditions. Inflammation, increased sympathetic tone and catecholamine surge in response to brain injury may affect different organs simultaneously, leading to their failure.

Acute brain injuries pose a great threat to global health as stroke stands out as the second most common cause of death overall, while traumatic brain injury is the leading cause of death and disability before age of 45 [1,2]. Cerebrovascular injury and brain trauma may differ in the origin of the primary damage, but the pathophysiological cascade that follows both of those conditions is similar, as the main consequences of the brain insult are: cerebral oedema, intracranial hypertension and cerebral vasospasm [3]. Patient with acute brain injury may develop distant organ failure even if no systemic diseases or infection are present (Figure 1). However, pathophysiological rationale suggests that in patients with prior organ insufficiencies, e.g., chronic kidney disease, chronic obstructive pulmonary disease and chronic heart disease, where biological reserves of such organs are reduced, any insult regarding central nervous system (CNS) could augment already present disease and worsen the course of its failure. Nevertheless, the severity of non-neurologic organs’ dysfunction depends on the extremity of brain injury. Additionally, dysfunction of non-neurologic organs can even further harm CNS. Therefore, the systemic manifestations of acute brain injuries impact mortality and disability. In this comprehensive review we sought to describe the organ-related consequences of acute brain injuries.

## 2. Endocrine Manifestations of Brain Injury

### 2.1. Pituitary Dysfunction

Brain injury have been associated with an impairment in pituitary secretion for many years. Greater severity of brain damage has been associated with higher risk of pituitary dysfunctions [4,5,6,7]. Post-traumatic hypopituitarism (PTHP) has been observed to occur in as many as 25–50% of patients after traumatic brain injury (TBI) [6,8,9], which would make TBI the most common cause of acquired hypopituitarism [10].

Some kind of pituitary dysfunction has been observed in up to 82% of patients with ischemic stroke [11] and 47% of patients with subarachnoid haemorrhage [12]. The most common presentation of this would be the growth hormone deficiency (25%) followed by hypogonadism (14%), hypothyroidism (10–17%), secondary adrenal deficit (8.5%) and prolactin altered levels (most commonly high values), although more than one hormone deficiency may occur at the same time [7,9,11,13,14,15]. PTHP should be assessed carefully as it may increase mortality in acute brain injury [16,17,18]. Is has been noticed that pituitary deficiency can occur in acute phase of TBI or even cause chronic endocrinopathies [19,20].

The pathophysiology of pituitary deficit in brain injury is multifactorial. Direct damage to the pituitary gland, infundibulum or hypothalamus may be a consequence of the head trauma [21]. Another theory suggests an ischemic insult as a primary pivot in the pathogenesis of PTHP [22]. This may originate from ischemic stroke per se or secondary from hypotension, hypoxia, anaemia and brain oedema accompanying acute settings of brain injury [22,23].

As pituitary deficiency is an emerging problem, a clinician who deals with patients with acute brain injury should be aware of possible signs and symptoms of pituitary deficiency (Table 1). Although TSH (thyrotropin), GH (growth hormone), PRL (prolactine) and gonadotropins’ levels alteration do not pose a threat in acute care, adrenal insufficiency and antidiuretic hormone secretion abnormalities may impact critical care management. (e.g., with the need for higher dosage of vasopressors and adjustments to fluid therapy respectively). Therefore, pituitary deficit should be considered and accurately assessed (Table 1) whenever water-electrolyte imbalance, hypoglycemia and excessive hypotension occurs [24,25].

### 2.2. Antidiuretic Hormone Secretion Abnormalities

Antidiuretic hormone (ADH) is produced by hypothalamic neurons in the supraoptic and paraventricular nuclei and then transported along the axons connecting them to posterior pituitary gland [26]. Cerebral injury may lead to damage of hypothalamic neurons producing ADH, their axons or posterior pituitary gland, and thus leading to ADH deficiency—central diabetes insipidus [26]. This causes disability to concentrate urine and consequently polyuria, polydipsia, hypernatremia and dehydration. On the contrary, brain trauma can also cause the opposite effect—syndrome of inappropriate antidiuretic hormone secretion (SIADH). SIADH has been recognized as a common aetiology of hyponatremia in SAH, TBI and ischemic stroke patients, resulting in hyponatremia with no symptoms of water depletion [27,28]. Water-electrolyte imbalance in acute brain injury patients will be described in more details further in the text. 

### 2.3. Thyroid Hormone

Hypothyroidism occurs in 10–30% of patients following traumatic brain injury. In traumatic and cerebrovascular brain injury peripheral conversion of tetraiodothyronine (T4) to triiodothyronine (T3) is reduced and thus low levels of T3 and a rise in reverse T3 (rT3) is observed [15,29,30]. This disorder is known as euthyroid sick syndrome [31]. These changes usually normalize after recovery but they are probably associated with poor outcome [32,33,34]. Central hypothyroisim (CH) occurs in 5–15% of TBI and it can be caused by altered secretion of thyrotropin (TSH) or TSH-releasing hormone (TRH) [15,35]. It may result from direct hypothalamic-pituitary axis (HPA) injury, increased sympathetic tone and high catecholamine release, which has been shown to associate with poor outcome [36]. The most common finding are low total T3 and T4 levels, however TSH levels depend on the phase of the critical illness – usually low levels early in the acute phase and higher than normal levels during the recovery [37]. There is no data supporting implementation of thyroid treatment in euthyroid sick syndrome, since it should be seen as adaptive mechanism [38]. Patients with hypothyroidism that occurred before the cerebral incident may develop myxoedema coma and then will require levothyroxine treatment [37].

### 2.4. Adrenal Cortex Hormones

Adrenal hormone reduction is estimated to occur in 15% of patients with TBI [15]. It may develop as a consequence of primary failure of the adrenal gland caused by systemic inflammatory response or secondarily to hypothalamic-pituitary axis (HPA) damage [39]. Adrenal hormones deficiency is clinically significant with hyponatremia, hypotension and hypoglycemia and thus monitoring cortisol level in acute brain injury patients should be considered [40]. Normally morning cortisol level is recommended, however in patients with acute conditions such as SAH or TBI circadian variations in serum cortisol are less evident [40]. It should be noted that adrenal insufficiency may be present in the chronic phase of recovery [41]. If clinical presentation suggests adrenal insufficiency, stimulation test with 250 μg of cosytropin may be considered to evaluate adrenal function, nonetheless in patients with direct injury of HPA this test may appear normal [37]. However, stimulation test is not a gold standard method. Glucocorticoid replacement should be considered in any type of proven adrenal insufficiency, whereas mineralocorticoids (such as fludrocortisone) may be implemented in primary adrenal insufficiency and is not needed in HPA injury [37,39]. It is worth mentioning that fludrocortisone use is rising, as it is used to manage hyponatremia in TBI and improve outcomes in sepsis [40,41].

Endocrine manifestations in acute brain injury are summarized in Table 1.

## 3. Heart

The link between brain and heart has been known since the 20th century, when Harvey Williams Cushing observed correlation between cerebral compression and blood pressure [50]. Cardiac dysfunction in brain injury is a relatively common and clinically significant condition. It is noticed to occur in 13–74% patients with traumatic brain injury (TBI) [51,52,53,54,55]. According to recent studies, 69% of patients with SAH present with an abnormal admission ECG [52]. Researchers found that markers of cardiac damage are associated with an increased risk of death, poor outcome and delayed cerebral ischemia after SAH [56,57]. Zygun et al. in their investigation of patients with severe TBI found that respiratory failure was the most common non-neurologic organ system failure, occurring in 23% of patients, whereas cardiovascular failure occurred in only 18% [58]. Neurogenic heart dysfunction has been recorded in multiple acute neurological conditions, such as subarachnoid haemorrhage (SAH) intracerebral haemorrhage, traumatic brain injury (TBI) and acute ischemic stroke (AIS). Among those, SAH seems to be of greater risk value [54,59,60,61,62,63,64]. It seems that the degree of neurological injury may be related to the severity of cardiac dysfunction. [65]. Collectively, clinical evidence suggests that the pathophysiology of cardiac dysfunction and brain injury intertwines.

**Figure 2:**
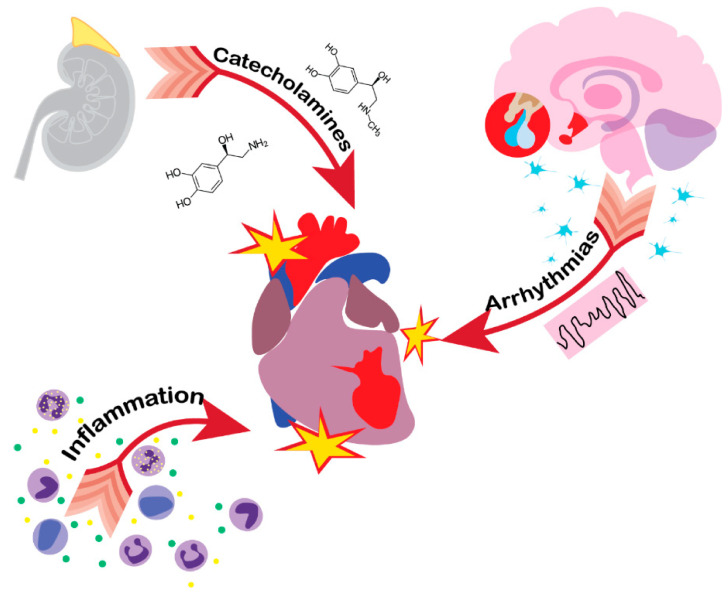
Pathological pathways of heart injury in acute brain damage patients. Catecholamine surge, inflammation and HPA axis imbalance are the leading causes of heart dysfunction following brain injury, which may manifest in Tako-tsubo cardiomyopathy.

The presumed aetiology of post-brain injury cardiac complications includes a wide variety of possible mechanisms of which some may be of more importance than the others (Figure 2). Throughout the literature, the catecholamine surge theory has been described the most frequently. It has been observed that brain injury causes an increase in catecholamine secretion, which is associated with worse outcome [66,67,68,69,70]. In addition to the rise of circulating catecholamines, local release of norepinephrine from myocardial nerve endings occurs [71,72]. Catecholamines may lead to cardiac injury in many mechanisms, including direct cytotoxicity, increasing myocardial demand and vasospasm of the coronary vessels [73,74].

The norepinephrine-induced toxic effect appeared to be the result of cyclic AMP-mediated calcium overload of the cell and a decrease in cardiocyte’s viability [75]. The end result of this is mitochondrial dysfunction and cell death at myocardium. Moreover, catecholamines may lead to structural changes in cardiomyocytes known as “contraction band necrosis”, which are characterized by focal myocytolysis, myofibrillar degeneration, and irregular cross-band formation. Not only serum and cerebrospinal fluid (CSF) catecholamines but also sympathetic and parasympathetic impulsation serve a role in myocardial damage [64,70]. Reduction in cerebral blood flow (CBF) provokes autonomic response of vasomotor centre. Initially the activation of sympathetic centre increases norepinephrine release at nerve terminals, which is presented by rise in mean arterial pressure (MAP), heart rate and cardiac output. At this point, carotid baroreceptors are activated in response to high MAP and thus bradycardia of Cushing triad occurs [64]. The autonomic imbalance caused by central nervous system (CNS) dysfunction may lead to myocardial damage and dangerous arrhythmias, such as prolongation of the corrected QT (QTc), T-wave inversions and ST segment changes [70,76,77]. The effect on the heart is dependent on the location of the ischemic lesion. It has been noticed that incidents localized in the insular region of the brain are associated with adverse cardiac outcomes [51,78]. It seems that right insular lesions result in sympathetic overactivity and thus associate with higher mortality than other sites [51,79]. The life-threatening arrhythmias after acute brain injury may also be caused by hypothalamic-pituitary-adrenal (HPA) axis dysfunction [80,81]. Finally, distant organ damage, including the heart, may be caused by systemic inflammatory responses that follows acute brain injury [81,82].

Currently we distinguish two clinical manifestations of brain injury-related cardiac dysfunction: neurogenic stunned myocardium (NSM) and acute stress induced cardiomyopathy (Tako-tsubo cardiomyopathy; TTS). Whether these are different conditions or simply manifestations of the same pathomechanism is still up for debate [83,84].

Tako-tsubo cardiomyopathy (TTS) also known as apical ballooning syndrome or broken heart syndrome was initially described by Hikaru Sato in the 1990s [85]. It has been described as a transient cardiac dysfunction following stressful events with the absence of coronary artery disease, predominantly happening in postmenopausal women [86]. It has been named after Japanese term for octopus catcher pot, which resembles the appearance of the left ventricle dyskinesia during systole on transthoracic echocardiogram (TTE) [85]. Tako-tsubo-like cardiac abnormalities pattern following SAH has been noticed by Ako et al. in 2003 [87]. In TTS, a wide variety of ECG changes may occur, including sinus tachycardia, sinus bradycardia, ST-segment elevations or depressions, T-wave inversions, and prolonged QTc intervals [88]. Biomarkers of myocardial injury, such as creatine kinase, creatine kinase-MB and cardiac troponin, are elevated in most patients with TTS [89]. The peak troponin levels are typically less than one would see in a true myocardial infarction [90]. Plasma B-type natriuretic peptide (BNP) levels are usually higher in TTS than in STEMI, and the ratio of BNP to peak troponin levels may differentiate TTS from STEMI [91]. The classic and most common type of wall motion abnormalities seen in imaging studies is apical ballooning, but variants such as midventricular [92] and basal [93] wall motion abnormalities have been observed. In TTS, left ventricular wall motion abnormalities extend beyond the distribution of a single coronary artery supply area and thus systolic dysfunction appears ‘circular’ at speckle-tracking echocardiography [94]. Cardiac wall motion assessment may be facilitated by using intravenous ultrasound contrast agents. This could be especially useful in patients in whom coronary angiography (CAG) is not performed due to active bleeding or other comorbid conditions that may enhance the risk of CAG [95]. Potential complications of TTS, such as LVOT (left ventricular outflow tract obstruction), acute mitral regurgitation, right ventricular (RV) involvement or apical thrombus, should always be considered and appropriately assessed [89].

Neurogenic stunned myocardium (NSM) is defined as cardiac abnormalities that are caused by neurological events such as traumatic brain injury, ischemic stroke or subarachnoid haemorrhage. It can cause electrocardiogram (ECG) abnormalities suggesting ischemia, wall motion dysfunction and raised troponin levels. Unlike myocardial infarction (MI) there is no significant obstruction of coronary vessels, although differentiating those two conditions can be difficult. NSM is very similar to Tako-tsubo cardiomyopathy in clinical presentation and pathophysiology. Both of those conditions are reversible [96] and like Tako-tsubo, NSM can cause changes suggestive of ischemia in ECG, raised troponin levels and wall motion abnormalities [97]. However, the differential factor of those two conditions would be the scope of cardiac wall hypokinesis, which is global in neurogenic stunned myocardium and regional in Tako-tsubo cardiomyopathy [96]. Whether one develops TTS or NSM could possibly depend on catecholamine release pattern [98]. It seems that in TTS epinephrine has been considered to play a pivotal role since apical myocardium has the greatest density of beta-adrenoreceptors [99]. By contrast, norepinephrine has been considered to be the main causative agent in the development of NSM, in which a greater raise in plasma catecholamine levels have been observed [100]. This is all the more important as norepinephrine is a commonly used agent in Intensive Care Unit (ICU) to maintain mean arterial pressure (MAP), especially in SAH patients.

Cardiac dysfunction is exceptionally relevant in patients with acute brain damage as it may result in a reduction of the left ventricular ejection fraction (LVEF). This may lead to hypotension, low cardiac output and decrease in cerebral blood flow (CBF), causing poor cerebral perfusion, further worsening patient’s outcome. Proper personalized haemodynamic management, including implementation of vasopressors, fluid administration and inotropic support, according to Frank-Starling law, should help maintain CBF.

It is worth mentioning that patients with baseline cardiovascular disease such as: hypertension, diffuse arteriosclerosis, heart failure or atrial fibrillation are prone to cerebrovascular incidents. Moreover, cardiac events may lead to falls, especially in the elderly, and thus result in traumatic brain injury. Both, predisposing and triggering factors for low CBP will negatively influence the heart and the brain, leading to negative outcome.

## 4. Lungs

Neurogenic pulmonary oedema (NPE) is a form of respiratory distress that timewise correlates with an onset of acute brain injury and cannot be explained by either heart failure or fluid overload [101]. NPE can occur in several conditions, such as: subarachnoid haemorrhage (SAH), intracerebral haemorrhage, trauma brain injury (TBI), status epilepticus, subdural hematoma, epidural hematoma and other CNS acute states [102,103,104,105,106,107,108,109,110]

All of the abovementioned diseases, despite different aetiologies, share some of the key elements for the development of NPE. Brain injuries that cause NPE are often expressed as an acute increase in the intracranial pressure (ICP), followed by decreased cerebral perfusion or a direct damage of brainstem and hypothalamus [111].

Such stress to which CNS is subjected can cause severe dysregulation of catecholamine homeostasis and lead to a massive α-adrenergic activation which is considered to be the key factor in the development of NPE [112]. Catecholamine storm causes a massive vasoconstriction which leads to an increased blood pressure and generates massive blood shifts, from systemic to pulmonary vasculature [113]. Those factors account for a critical elevation of hydrostatic pressure within pulmonary capillaries which not only causes fluid leakage but also damages endothelium [114]. However, other mechanisms leading to NPE are also discussed, since respiratory failure can occur in patients in whom no increase in systemic pressure was observed [115]. Some experimental studies showed that denervated lungs or sympathetic gangliotomy protect lungs from suffering from NPE which would indicate direct neural mediation of such response [116,117]. Additionally, brain injury can lead to a systemic inflammation, with a number of proinflammatory cytokines being released which increase permeability of pulmonary capillaries, promoting lung edema [118].

NPE manifests as a typical respiratory failure. Patients often exhibit dyspnoea, tachypnoe, cyanosis, produce frothy sputum and the lungs are often bilaterally infiltrated in chest X-ray imaging [101]. Two distinct phenotypes of NPE can be distinguished. First phenotype, the fulminant one, occurs within 30–60 min after brain injury and is associated with significantly higher mortality than the second one which occurs within 12–24 h after injury (>60% vs. ~50%) [119]. In ~50% of all cases, NPE resolves within 72 h after onset [120]. The frequency of NPE and its severity seem to be dependent on the severity of brain injury. In one study, NPE occurred in 8% of aneurysmal SAH patients. However, the percentage of NPE increased in patients with more severe SAH (grade IV and V), resulting in 31% in this subgroup [102]. In this study cohort, NPE was associated with 95% mortality. Such a high mortality of patients with NPE would then be explained by the fact that it is correlated with severity of the brain injury. Post-mortem analysis of patients who experienced acute brain injury showed that more than 70% of all patients experience some kind of pulmonary edema which is not necessarily clinically significant [121]. The presence of NPE can additionally further harm the brain, as hypoxia leads to inadequate oxygen supply to the brain cells and hypercapnia can lead to cerebral vasodilation which increases cerebral blood flow and, therefore, can worsen the course of brain edema associated with trauma. The treatment of NPE is mostly supportive and consists of implementing: protective mechanical ventilation, adequate fluid balance (often fluid reduction) and if necessary, vasopressor drugs [114].

## 5. Kidneys

In order to understand the renal response to brain damage, one needs to realize that it does not always result in kidney injury [122]. Most disturbances that occur within those organs are often a result of antidiuretic hormone (ADH) imbalance secretion, which often manifest as hyponatremia (<135 mmol/L) [123]. The reported incidence of hyponatremia in TBI ranges from 9.6–51% and is the most frequent electrolyte disturbance in neurocritical care setting [40,124]. This can be expressed as either syndrome of inappropriate antidiuretic hormone secretion (SIADH) or cerebral salt-wasting syndrome (CSWS). In normal conditions, ADH secretion is stimulated by plasma hyperosmolality, however, in cases of brain injury, such secretion may be sustained without the natural, physiological trigger. Increased concentrations of ADH stimulate V2-receptors located in the basolateral membrane of collecting ducts in kidneys and lead to excessive water reabsorption and highly concentrated urine production [125]. Such positive free water balance and natriuresis manifest as hyponatremia [122]. In CSWS, the suggested pathomechanism is different. The main distinction between this disease and SIADH is fluid balance. Although both of these syndromes cause hyponatremia, patients with CSWS produce large amounts of urine, in contrast to patients with SIADH [126,127]. One of the speculated pathomechanisms for the CSWS development is an uncontrolled release of natriuretic peptides. Berendes et al. found that patients with SAH have much higher brain natriuretic peptide (BNP) concentrations than controls [127]. BNP leads to natriuresis that is followed by excretion of water [27]. CSWS patients, therefore, are hypovolemic and hyponatraemic. Nevertheless, some researchers question the need to distinguish SIADH and CSWS, as no therapeutic differences exist - both of those disorders are treated with the infusion of hypertonic saline and restriction of sodium-free water intake is recommended [122,128]. The presence of SIADH and CSWS could additionally worsen the course of acute brain injury. Hyponatremia and subsequent hypoosmolality harms the brain as it disturbs electrolyte balance within brain tissue and works as a factor in promoting brain edema.

Another disruption of electrolyte-water balance in brain injury is hypernatremia (>145 mmol/L) [129]. Many CNS acute conditions can lead to posterior pituitary gland dysfunction that lead to a decreased ADH secretion [26]. The cause of such dysfunction can be a result of direct damage to pituitary gland or vascular injury that occurs in TBI [130,131]. This manifests as diabetes insipidus (DI) which is expressed as polyuria, low urine osmolality and hypernatremia [132]. The prevalence of DI after acute brain injury is difficult to assess, ranging from 2.9% to 51% (in TBI) [133,134]. It is additionally, highly associated with mortality, as 57% to 69% of brain injury associated-DI patients die [135]. It must be remembered that hypernatremia does not need to derive only from CNS dysfunction in neurocritical care setting. Actually, many of hypernatremia cases result from iatrogenic interventions [122]. Those include water loss due to mannitol administration, infusion of hypertonic saline, inadequate fluid therapy and administration of loop diuretics [122].

Catecholamine surge, as described in the “heart” section of this review, leads to an increased cardiac output and elevated blood pressure. This, naturally, causes renal blood hyperperfusion which results in augmented creatinine clearance [136]. Udy et al. showed that for those patients, norepinephrine use, saline loading, mean arterial blood pressure and central venous pressure were associated with elevated GFR. Additionally, they found that in TBI patients, atrial natriuretic peptide (ANP) concentrations were also increased which could additionally connect high GFR values with the release of natriuretic peptides [137].

All of the abovementioned disorders derive solely from CNS injury, while renal function remains intact. However, in some cases, as mentioned in other sections of this review, brain injury induces systemic inflammatory response and may result in pro-inflammatory cytokine storm that damages renal tubular epithelium and microvasculature in a similar way as in sepsis acute kidney injury (AKI) [122]. Additionally, the kidneys are highly perfused organs with low vascular resistance which can affect their function during sudden upstream changes of blood pressure, leading to organ injury [138]. Moreover, the crosstalk between heart and brain, as described in the upper section of this review, indicates that secondary damage to the heart impairs myocardial contractility, leading to decreased cardiac output. This causes kidney hypoperfusion and therefore, a risk of AKI. Such renal impairment may even require implementation of continuous renal replacement therapy which may additionally attenuate the levels of proinflammatory cytokines [139].

## 6. Immune System

There is a constant interplay between CNS and the immune system in acute brain injury. Leukocyte infiltration of the brain through the blood-brain-barrier (BBB) has been largely documented and can both attenuate and worsen neurological prognosis [134]. However, extracerebral consequences of the immune system activation are of clinical importance and influence mortality. Indeed, increased susceptibility to infections in neurocritically ill patients has been numerously reported [2,140,141,142].

Acute distress that occurs within the CNS, as previously described, often initiates a wide array of neuroendocrine and neurotransmitter disruptions. Excessive HPA axis and sympathetic nervous (SNS) system activation have a significant effect on the spleen as 98% of all splenic innervation is of SNS origin [143]. There have been reports that after ischaemic and haemorrhagic stroke, a spleen contraction occurs, resulting in peripheral immune activation: leukocyte (mainly neutrophils) and pro-inflammatory cytokines level (IL-1, IL-6, TNF-a etc.) increase [140,144,145]. This is known as an acute inflammatory phase and takes place 6–22 h after brain injury and may be associated with severe inflammatory response syndrome (SIRS) [146,147]. Then, within 4 days, subacute immune-suppression occurs and a number of anti-inflammatory cytokines are being released (e.g., noradrenaline stimulates IL-10 production) and T-reg cells level elevates [147,148,149,150,151]. Besides spleen shrinkage, macrophages, lymphocytes and NK-cells express β2-receptors which, when activated, promote their apoptosis and reduce cell proliferation [148,152,153].

It becomes clear that increased susceptibility to infection in acute brain injuries take place during immunosuppression phase [151]. Indeed, pneumonia is the main cause of death in patients after stroke [151]. It is, however, hypothesised that such immune depression may play an adaptive role in surviving CNS injury, as due to BBB disruption, CNS-specific antigens are released to systemic circulation and are exposed to immune response. This is speculated to induce autoimmune response and worsen the course of brain damage due to autoaggression [151,154,155]. Therefore, the immunosuppression phase could attenuate the latter effect.

## 7. Digestive Tract

One of the possible gastrointestinal manifestation of brain injury could be Cushing’s ulcer, also known as stress ulcer. As its prevalence in severely injured patients is high, gastroduodenal complications are of great significance in intensive care unit (ICU) settings [156,157,158]. The pathophysiology behind gastro-duodenal ulceration connected to brain disease is still not fully understood. The injury of hypothalamic-pituitary axis results in release of corticosteroids that are known to change the constitution of gastric mucus and impair mucosal regeneration. [159]. However, parasympathetic stimulation pathway leading to ulceration seems to be more likely. With acute brain injury intracranial pressure (ICP) rise, similarly to brain tumours, gastroduodenal ulcers occur [160]. Damage of the hypothalamus may result in destruction of sympathetic centres, leading to overstimulation of vagus nerve and hyperacidity [157]. It is worth mentioning that damage to the gastric mucosa may happen as a result of conditions secondary to brain injury such as shock, and acid-base imbalance [161]. Options for pharmacological prophylactic agents include proton pump inhibitors (PPIs), histamine_2_-receptor antagonists (H_2_Ras) and sucralfate, a mucosa-protective agent acting as acid buffer [162]. Historically, PPIs were thought to be more effective in reducing gastrointestinal bleeding, but it has been observed lately that not only do they not surpass H2Ras, but also may cause dangerous hypomagnesemia and increase mortality [163,164,165]. Although acid-suppressing therapy is implemented commonly in the ICU settings, recent studies suggest that it is not beneficial in most patients and the adverse effects in the form of pneumonia and *Clostridium difficile*-associated diarrhea contradict its profitability [166,167,168]. However, non-pharmacological prophylaxis with early enteral nutrition may increase gastrointestinal blood flow and oxygenation, which can potentially contribute to recovery [169,170]. In TBI, immunonutrition, which is mostly implemented in surgical patients, may possibly reduce cytokines, increase antioxidant indices and reduce infection complications [171].

The interactions between central nervous system (CNS) and gastrointestinal (GI) tract has been observed for years now. The GI is controlled by autonomic nervous system, but it is also affected by HPA axis. The so-called brain-gut axis may be responsible for increased permeability of the ileal and colonic wall. This may result from brain tissue secretion of proinflammatory molecules and thus cause the release of bacteria and toxins from intestinal lumen into circulation [172]. This condition is known as “leaky gut” and may lead to SIRS or even to sepsis due to secondary bacteraemia [173,174].

## 8. Conclusions

When dealing with brain injury patient, one usually focuses on the cerebral insult itself. With this review, the authors wanted to emphasize that central nervous system damage may manifest in wide variety of distant organs. It’s vital to understand that critical care pathways used to treat TBI may also influence distant organ dysfunction. For example, osmotic therapy may cause serious abnormalities in water-electrolyte balance [175], which in turn would be detrimental to the kidneys and the gut. Acute respiratory failure promotes organ ischaemia and dysfunction. Also, mechanical ventilation may induce lung injury and reduction of cardiac preload. Additionally, those patients are at risk of infections and sepsis. The clinician should always be aware of the interplay between CNS and non-neurological organs, that is constantly present. It’s multidirectional and complex. The role of primary susceptibility of individual patients due to co-morbidities and frailty cannot be neglected. Cerebral injury is not only a brain disease, but also affects the body as whole, and thus requires holistic therapeutical approach.

## Figures and Tables

**Table 1:** Endocrine manifestations in acute brain injury [15,19,28,38,42,43,44,45,46,47,48,49].

Hormone/-s			Methods of Assessment	Signs and Symptoms in the Acute Phase	Signs and Symptoms in the Chronic Phase	Need of Replacement Therapy
**Antidiuretic Hormone**						
		SIADH	hyponatremia (<135 mmol/L) urine osmolality > 100 mOsm/kg	Oliguria	As in the chronic phase	-
		Central DI	hypernatremia (>145 mmol/L) urine osmolality < 250 mOsm/kg H_2_O low serum vasopressin levels	Polyuria Polydypsia	As in the chronic phase	If central DI is present—vasopressin replacement therapy with desmopressin
Growth hormone (GH)			Levels of GH and insulin-like growth factor 1 (IGF-1), GH response after growth hormone-releasing hormone (GHRH)	Not clinically evident	Poor quality of life, low mood, fatigue, cognitive dysfunction, reduction in muscle mass, increased body fat, decreased exercise capacity, increased lipid levels, reduced body mineral density, reduced LV mass, impaired LVEF	May partially reverse cognitive dysfunction after TBI.
Adrenal hormones (mineralocorticoid and glucocorticoid)			Serum cortisol concentration (<15–18 μg/dL/413–497 nmol/L), ACTH stimulation test (a change ≥9 μg/dL/248 nmol/L—adequate response)	Greater requirements for vasoactive therapy, hypoglycemia, hyponatremia, relative or absolute hyperkalaemia, rapidly progressive hypotension with hyperdynamic cardiovascular response with low SVR	Recurrent infections, fatigue, weight loss, nauseas, vomiting, hypoglycemia (mostly fasting), anorexia, myalgia Adrenal crisis: hypotension, hyponatremia, hypercalcemia, hyperkalaemia, syncope	Hormonal replacement therapy should be provided if hypoadrenalism have been confirmed. Hydrocortisone should be administered in all cases of adrenal failure, however mineralocorticoid supplemental is recommended if primary failure occurs.
TSH, T3, T4			Level of total serum T3 and T4, TSH concentration	May resemble symptoms of already acutely ill patients: impaired consciousness, myocardial dysfunction, hypothermia, neuropathy, muscle weakness, skin atrophy	Dry skin, excessive weight, bradycardia, systemic hypertension, fatigue, constipation, cold intolerance, muscle aches, vocal changes, prolonged ankle-jerk reflex time, hyponatremia	

SIADH—syndrome of inappropriate antidiuretic hormone hypersecretion DI—diabetes inspidius, LV—left ventricle, LVEF—left ventricle ejection fraction, SVR—systemic vascular resistance.
